# Delineation of molecular pathway activities of the chronic antidepressant treatment response suggests important roles for glutamatergic and ubiquitin–proteasome systems

**DOI:** 10.1038/tp.2017.39

**Published:** 2017-04-04

**Authors:** D I Park, C Dournes, I Sillaber, M Ising, J M Asara, C Webhofer, M D Filiou, M B Müller, C W Turck

**Affiliations:** 1 Department of Translational Research in Psychiatry, Max Planck Institute of Psychiatry, Munich, Germany; 2 Department of Stress Neurobiology and Neurogenetics, Max Planck Institute of Psychiatry, Munich, Germany; 3 Phenoquest AG, Martinsried, Germany; 4 Department of Clinical Research, Max Planck Institute of Psychiatry, Munich, Germany; 5 Division of Signal Transduction, Beth Israel Deaconess Medical Center, Boston, MA, USA; 6 Department of Medicine, Harvard Medical School, Boston, MA, USA; 7 Division of Experimental Psychiatry, Focus Program Translational Neuroscience, Department of Psychiatry and Psychotherapy, Johannes Gutenberg University Medical Center, Mainz, Germany

## Abstract

The aim of this study was to identify molecular pathways related to antidepressant response. We administered paroxetine to the DBA/2J mice for 28 days. Following the treatment, the mice were grouped into responders or non-responders depending on the time they spent immobile in the forced swim test. Hippocampal metabolomics and proteomics analyses revealed that chronic paroxetine treatment affects glutamate-related metabolite and protein levels differentially in the two groups. We found significant differences in the expression of *N*-methyl-d-aspartate receptor and neuronal nitric oxide synthase proteins between the two groups, without any significant alterations in the respective transcript levels. In addition, we found that chronic paroxetine treatment altered the levels of proteins associated with the ubiquitin–proteasome system (UPS). The soluble guanylate cyclase-β1, proteasome subunit α type-2 and ubiquitination levels were also affected in peripheral blood mononuclear cells from antidepressant responder and non-responder patients suffering from major depressive disorder. We submit that the glutamatergic system and UPS have a crucial role in the antidepressant treatment response in both mice and humans.

## Introduction

The high non-response rate to antidepressant treatment is a major problem in clinical practice. Over one-third of major depressive disorder (MDD) patients do not achieve full remission, experiencing symptom recurrence despite antidepressant treatment.^1, 2, 3^

Although the exact mechanism of antidepressant response remains unknown, numerous studies have tried to identify biological pathways as potential biomarkers for antidepressant treatment response. Serotonin transporter^4^ and serotonin autoreceptors^5^ have been found to be critical for the antidepressant response. In addition, alterations and abnormalities of the hypothalamus–pituitary–adrenal axis have been associated with antidepressant treatment outcome.^6^ The brain-derived neurotrophic factor gene *Val66Met* polymorphism has also been studied with regard to the antidepressant treatment response and was shown to result in antidepressant treatment resistance in rodents and humans.^7, 8^ A link between inflammatory cytokines and antidepressant response has been documented. Cerebrospinal fluid interleukin-1, interleukin-6 and tumour necrosis factor-α blood levels in MDD patients were significantly correlated with depression severity.^9^ High cytokine concentrations have been found in antidepressant treatment-resistant depression patients.^10, 11, 12^

Fast-acting antidepressant-like agents have also been investigated illuminating novel molecular pathways associated with antidepressant response. Scopolamine,^13^ an antagonist for muscarinic cholinergic receptors, and ketamine,^14^ an *N*-methyl-d-aspartate receptor (NMDAR) antagonist, have a rapid antidepressant-like effect in treatment-resistant depression patients. Low doses of ketamine were found to increase glutamate transmission that mediates brain-derived neurotrophic factor synthesis and synaptogenesis that are important for antidepressant-like action.^15^

Our previous studies have shown that energy metabolism pathways may be associated with antidepressant response. Acute ketamine treatment resulted in significant energy metabolism changes.^16^ This was also evidenced by hippocampal glycogen and energy metabolism alterations in chronic paroxetine-treated mice.^17^

In the current study, paroxetine-treated DBA/2 J mice that were previously shown to respond to chronic paroxetine treatment^18^ were grouped into responders or non-responders based on their forced swim test (FST) behaviour. Quantitative proteomics and metabolomics analyses were used to identify antidepressant response-associated pathways in brain samples. The relevance of the identified pathways was examined in peripheral blood mononuclear cells (PBMCs) taken from patients diagnosed with MDD.

## Materials and methods

Details for mouse brain and blood collection, proteomics and metabolomics analyses, functional enrichment analysis, western blot analysis, quantitative reverse transcription PCR and immunoprecipitation are provided as a part of ‘Supplementary Information'.

### Animal housing and husbandry

The experiments were carried out with male DBA/2 J mice (Charles River Laboratories, Chatillon-sur-Chalaronne, France). All the animals were between 8 and 10 weeks old and single housed for at least 1 week before the beginning of the experiments. The mice were kept under normal light and temperature conditions (12 light: 12 dark light cycle, lights on at 1900 h, temperature maintained at 23±2 °C and humidity at 55±5%) with standard bedding and nesting material, in polycarbonate cages (21 × 15 × 14 cm). Water and Altromin 1324 standard mouse chow (Altromin, Lage, Germany) were provided *ad libitum*. All the procedures were carried out in accordance with the European Communities Council Directive 2010/63/EU and approved by the committee for the Care and Use of Laboratory animals of the Government of Upper Bavaria, Germany.

### Drug administration

The mice were treated with vehicle or 5 mg kg^−1^ paroxetine pills (Paroxetine hydrochloride; Carbone Scientific, London, UK) for 28 days, twice a day. The animals were randomly assigned to the vehicle- or paroxetine-treated experimental group. Vehicle or paroxetine was administered via customized palatable pellets (40 mg PQPellets, Phenoquest, Martinsried, Germany). To control for environmental effects, such as social stress between group-housed male mice, all the mice were single housed during vehicle and paroxetine treatment to ensure accurate dosing. The animals that did not take the pellets properly were excluded from further analysis.

### Behavioural testing

#### Forced swim test

Each mouse was put into a glass beaker (height 24 cm, diameter 13 cm) that had been filled with 21±1 °C water up to a height of 15 cm. This meant that the animals were unable to touch the bottom or escape for 6 min testing period. Immobility time was measured for the entire 6 min test. The amount of time the mouse spent immobile was scored by an experienced observer who was blind to the experimental group.

### Patient samples

The PBMCs obtained from 17 participants of the Munich Antidepressant Response Signature study were included for assessing protein expression levels (Supplementary Table 4). All the 17 depressed patients were treated for 4–6 weeks with different types of antidepressant drugs that included tricyclic antidepressants, selective serotonin reuptake inhibitors, serotonergic and noradrenergic reuptake inhibitors, noradrenergic and selective serotonergic antidepressants, noradrenergic reuptake inhibitors and selective serotonin reuptake enhancers. Diagnosis was conducted according to DSM-IV criteria. Depression severity was evaluated using the 21-item Hamilton Depression Rating Scale. Responder patients were classified based on their clinical antidepressant treatment response corresponding to minimal 50% reduction in the Hamilton Depression Rating Scale score between baseline and after 6 weeks of treatment. The Munich Antidepressant Response Signature project was approved by the ethics committee of the Medical Faculty of the Ludwig Maximilians University Munich, Germany (submission number 318/00). Participants included in the study gave oral and written consents.

### Hierarchical clustering analysis

Hierarchical clustering analysis is a method to build and split different cluster hierarchies. It has been applied to identify subgroups of cells and animals based on marker protein expression or behavioural parameters.^19, 20^ Hierarchical clustering analysis was carried out with SPSS (SPSS version 21, IBM SPSS, Chicago, IL, USA) to separate vehicle- or paroxetine-treated mice into subgroups. Based on FST immobility time, three subgroups that include long-time floating, intermediate-time floating and short-time floating groups were stratified for each treatment condition (vehicle or paroxetine).

### Statistical analysis

Statistical analyses of the FST behavioural data and covariates were performed with GraphPad Prism 5 (GraphPad Software, La Jolla, CA, USA). The *t*-test or one-way analysis of variance were used to assess statistical significance between groups. For the identification of significantly altered metabolites, metabolite peak intensities were median and auto-scaled normalized. Metabolites with missing values, 30 for the hippocampus in all replicates, were excluded from data analysis. Metabolite level differences between paroxetine-treated long-time floating (PLF) and paroxetine-treated short-time floating (PSF) groups were calculated using *t*-test (*P*<0.05), followed by false discovery rate correction (*q*<0.1). Proteomics data were corrected for multiple testing by Benjamini–Hochberg.

Western blot data were also analysed with GraphPad Prism 5. One-way analysis of variance with Tukey's test was used to evaluate the protein level difference among the groups. Spearman correlation (*r*) was used to assess the correlation of proteins with FST immobile time of mice. Pearson's correlation (*r*) was used to evaluate the correlation of proteins with clinical antidepressant response in depressed patients. Data were expressed as the mean±s.e.m. Statistical data were considered significant at *P*<0.05. To check normal distribution of data, D'Agostino's normality test was used. Sample size was determined based on our previous results.^17, 21^

## Results

### Identification of protein–metabolite network related to heterogeneous antidepressant response in mice

**Figure 1:**
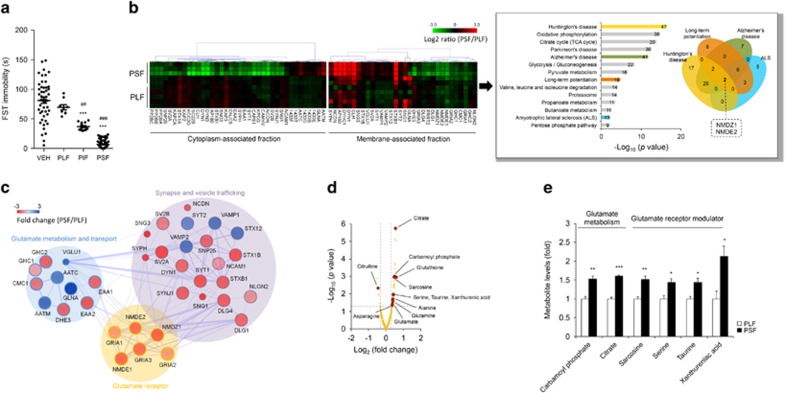
Identification of hippocampal protein–metabolite networks associated with the heterogeneous antidepressant treatment response in mice. (**a**) Paroxetine-treated mice were categorized into paroxetine-treated long-time floating (PLF), paroxetine-treated intermediate-time floating (PIF) and paroxetine-treated short-time floating (PSF) groups based on FST immobile time, *n*(VEH/PLF/PIF/PSF)*=*47/9/14/72. (**b**) Representative proteomics profiles and enriched pathways between the PLF and PSF groups. Proteomics profile differences between the two groups enriched several functional pathways. The shared protein signatures among the pathways were obtained using Venn diagram analysis. In the heat map, colours denote log_2_ ratio. Numbers in the enriched pathways indicates the number of proteins identified, *n*=5 per group. (**c**) Protein interaction network analysis based on proteomics data. In the interaction pathway map, colours denote fold difference between the two groups. Proteins with >20% fold change and adjusted *P*-value <0.05 were considered significant, *n*=5 per group. Designations for proteins in the heat map and protein interaction networks are based on Uniprot database. In the network, line thickness indicates the confidence of protein–protein interaction data. Small node represents protein with unknown three-dimensional (3D) structure. Large node represents protein with known or predicted 3D structure. (**d**) Volcano plot comparing PLF and PSF metabolomes. Metabolites with |log_2_(fold change)|>0.3 and –log_10_(*P*-value)>1.3 were considered significant, corresponding to >20% fold change and 0.05<*P*, *n*=5 per group. (**e**) Glutamate pathway-related metabolite differences between PLF and PSF mice, *n*=5 per group. Data are expressed as the mean±s.e.m. ***P<0.001 vs VEH, ^##^*P*<0.01 vs PLF, ^###^*P*<0.001 vs PLF and PIF (one-way analysis of variance (ANOVA) with Tukey's test, Figure 1a), *q<0.1, **q<0.05, ***q<0.001 (two-tailed *t*-test followed by false discovery rate (FDR) correction, Figure 1e). FST, forced swim test; VEH, vehicle.

The mice were treated with paroxetine (5 mg kg^−1^, twice a day) for 28 days. Three animal subgroups were identified according to FST immobile time (F_3,141_=132.1, *P*<0.0001; Figure 1a). To investigate the systemic effect of chronic paroxetine treatment on hippocampal molecular pathways, proteomic analyses were performed of the two extreme groups, PLF and PSF groups, which resulted in significant protein expression differences both in membrane- and cytoplasm-associated fractions (Figure 1b). GluN1 and GluN2B proteins were found to be shared by four enriched functional pathways related to amyotrophic lateral sclerosis, Alzheimer's disease, Huntington's disease and long-term potentiation. Based on this finding, we further investigated protein–protein interaction network using our proteomics data. The analysis showed extensive interactions among pathways related to glutamatergic transmission (Figure 1c). Hippocampal metabolite profiling data also showed altered levels of relevant glutamatergic receptor modulators and metabolites that are part of glutamate metabolism pathway (Figure 1d). After false discovery rate correction, several metabolites were at significantly higher levels in PSF compared with the PLF mice (Figure 1e).

**Figure 2:**
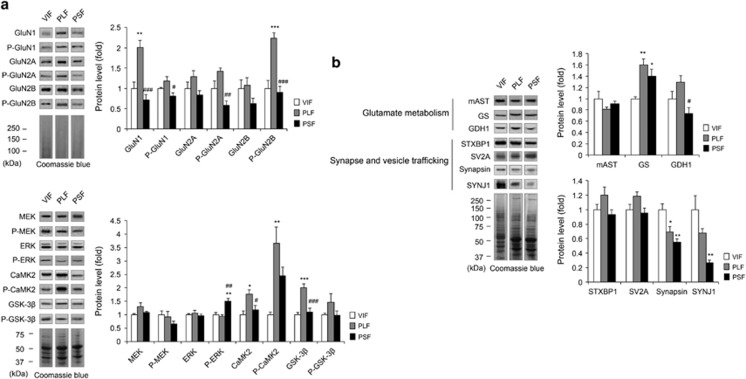
Chronic paroxetine treatment resulted in differential expression of glutamatergic pathway proteins between PLF and PSF groups. (**a**) NMDAR subunits and signalling protein and phosphorylation levels among the groups. The NMDAR subunit proteins were blotted using membrane-associated fraction. The NMDAR signalling proteins were blotted using cytoplasm-associated fraction, *n*=5 per group. (**b**) Glutamate metabolism and synapse/vesicle trafficking pathway protein level differences among the groups, *n*=5 per group. The proteins were blotted using cytoplasm-associated fraction. Data are expressed as the mean±s.e.m. *P<0.05, **P<0.01, ***P<0.001 vs VIF, ^#^*P*<0.05, ^##^*P*<0.01, ^###^*P*<0.001 vs PLF (one-way analysis of variance (ANOVA) with Tukey's test). Coomassie brilliant blue staining was used as a loading control. The VIF mice were selected as a control group. NMDAR, *N*-methyl-d-aspartate receptor; PLF, paroxetine-treated long-time floating; PSF, paroxetine-treated short-time floating; SYNJ1, synaptojanin 1; VIF, vehicle-treated intermediate-time floating.

Western blot analyses were performed to assess glutamatergic pathway protein level differences between the PLF and PSF mice (Figure 2 and Supplementary Figure 1). The GluN1, phospho-GluN1 (P-GluN1), phospho-GluN2A (P-GluN2A) and P-GluN2B levels were significantly different between the PLF and PSF groups (GluN1: F_3,16_=18.27, *P*<0.0001; P-GluN1: F_3,16_=3.43, *P*<0.05; P-GluN2A: F_3,16_=7.12, *P*<0.01; P-GluN2B: F_3,16_=17.30, *P*<0.0001; Figure 2a). GluN1 and phospho-GluN2B (P-GluN2B) proteins were significantly upregulated in PLF mice, whereas PSF mice did not show NMDAR subunit and phosphorylation level changes when compared with control group. Ca^2+^/calmodulin-dependent protein kinase 2 (CaMK2), phospho-CaMK2 (P-CaMK2) and glycogen synthase kinase-3β (GSK-3β) protein levels were significantly upregulated in PLF mice, whereas PSF mice exhibited a similar level of NMDAR signalling proteins except phospho-extracellular signal-regulated kinase (P-ERK), which were upregulated compared with the other groups. P-ERK, CaMK2 and GSK-3β levels showed significant differences between the PLF and PSF mice (P-ERK: F_2,12_=12.44, *P*<0.01; CaMK2: F_2,12_=6.62, *P*<0.05; GSK-3β: F_2,12_=19.75, *P*<0.001).

We also investigated proteins in the pathways of glutamate metabolism and synapse/vesicle trafficking (Figure 2b). Although chronic paroxetine treatment increased glutamine synthetase protein expression both in PLF and PSF mice, only glutamate dehydrogenase 1 (GDH1) level was significantly distinct between the two groups (F_2,12_=5.42, *P*<0.05). Synapsin and SYNJ1 (synaptojanin 1) protein expressions were downregulated by chronic paroxetine treatment without significant differences between the PLF and PSF mice.

**Figure 3:**
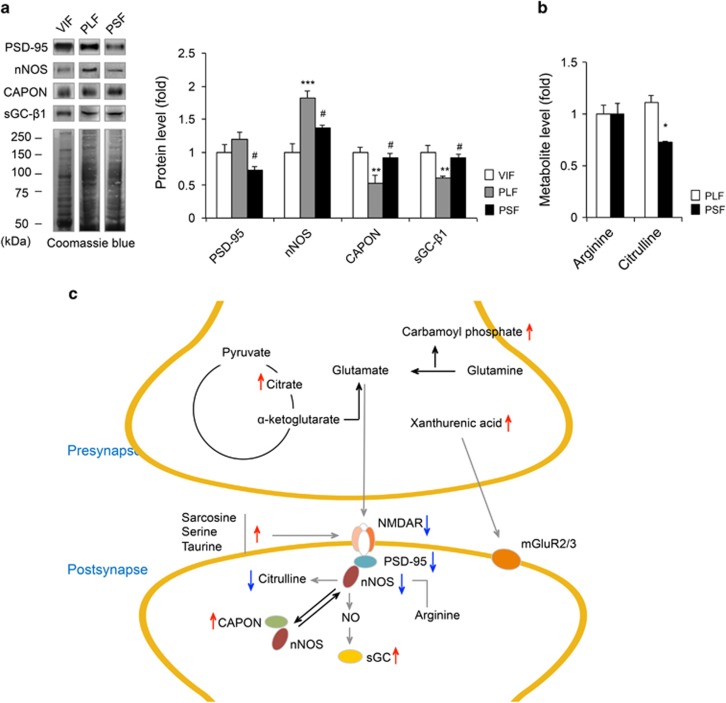
Differential effect of chronic paroxetine treatment on PSD-95/nNOS complex. (**a**) PSD-95, nNOS, CAPON and sGC-β1 protein level differences among groups, *n*=5 per group. The proteins were blotted using cytoplasm-associated fraction. (**b**) Arginine and citrulline levels in PLF and PSF mice, *n*=5 per group. (**c**) Affected protein–metabolite network following chronic paroxetine treatment. Upward-pointing red arrow indicates higher biosignature level in PSF compared to PLF mice. Downward-pointing blue arrow indicates lower biosignature level in PSF compared with PLF mice. Data are expressed as the mean±s.e.m. **P<0.01 vs PLF (two-tailed *t*-test), ***P<0.001 vs VIF, ^#^*P*<0.05 vs PLF (one-way analysis of variance (ANOVA) with Tukey's test, Figure 3a). *q<0.1 (two-tailed *t*-test followed by false discovery rate (FDR) correction, Figure 3b). Coomassie Brilliant Blue staining was used as a loading control. VIF mice were selected as a control group. CAPON, carboxy-terminal PDZ ligand of nNOS; mGluR2/3, metabotropic glutamate receptor 2 and 3; NMDAR, *N*-methyl-d-aspartate receptor; nNOS, neuronal nitric oxide synthase; PLF, paroxetine-treated long-time floating; PSD-95, postsynaptic density protein-95; PSF, paroxetine-treated short-time floating; sGC-β1, soluble guanylate cyclase-β1; VIF, vehicle-treated intermediate-time floating.

To determine whether chronic paroxetine treatment differentially regulated nitric oxide (NO) system in relation to glutamatergic pathway changes, we extended our study to investigate NO-related proteins and metabolites (Figure 3 and Supplementary Figure 2a). NO system-related proteins including postsynaptic density protein-95 (PSD-95), neuronal nitric oxide synthase (nNOS), carboxy-terminal PDZ ligand of nNOS (CAPON) and soluble guanylate cyclase-β1 (sGC-β1) showed significant level differences between PLF and PSF groups (PSD-95: F_2,12_=5.90, *P*<0.05; nNOS: F_2,12_=15.57, *P*<0.001; CAPON: F_2,12_=7.55, *P*<0.01; sGC-β1: F_2,12_=9.78, *P*<0.01; Figure 3a). Altered levels of citrulline whose conversion from arginine is catalysed by nNOS protein were detected (Figure 3b). Taken together, through integration of proteomic and metabolomic data, we were able to identify a systemic protein–metabolite network that is differentially affected between the PLF and PSF groups (Figure 3c).

### Chronic paroxetine treatment induces differential ubiquitin–proteasome system profiles

**Figure 4:**
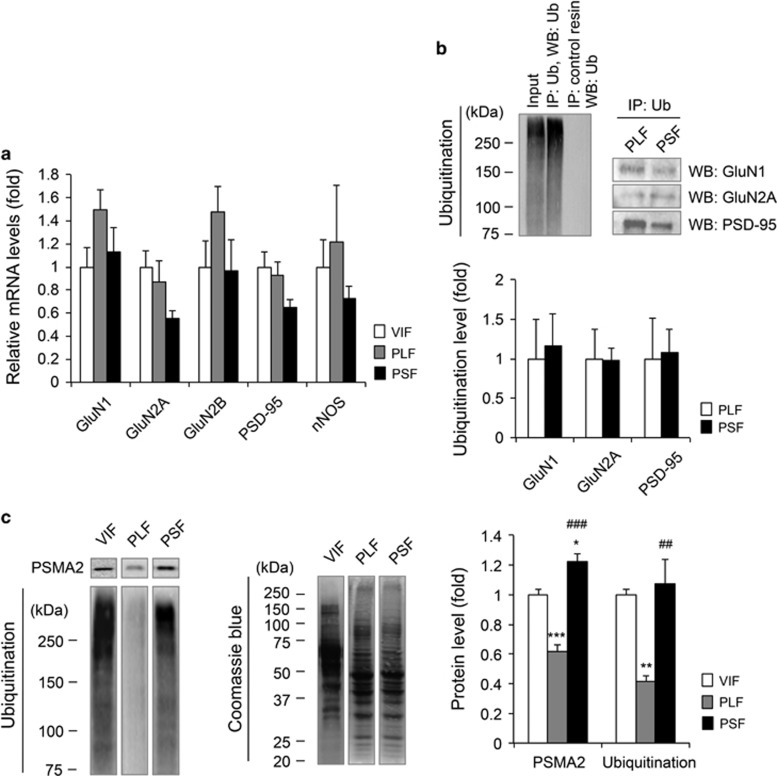
Differential effect of chronic paroxetine treatment on UPS. (**a**) Quantitative reverse transcription PCR data of NMDAR subunits, PSD-95 and nNOS showed no transcription differences between PLF and PSF groups, *n*=6 per group. (**b**) Immunoprecipitation (IP) study of ubiquitinated NMDAR subunits and PSD-95. IP with ubiquitin antibody was followed by western blot (WB) analysis of the proteins. No ubiquitinated protein level differences was observed between the PLF and PSF groups, *n*=3 per group. The proteins were blotted using total lysate extract. (**c**) PSMA2 and ubiquitination level differences between the PLF and PSF groups, *n*=5 per group. The proteins were blotted using cytoplasm-associated fraction. *P<0.05, **P<0.01, ***P<0.001 vs VIF, ^##^*P*<0.01, ^###^*P*<0.001 vs PLF (one-way analysis of variance (ANOVA) with Tukey's test). Coomassie brilliant blue staining is shown as loading control. VIF mice were selected as a control group. NMDAR, *N*-methyl-d-aspartate receptor; nNOS, neuronal nitric oxide synthase; PLF, paroxetine-treated long-time floating; PSD-95, postsynaptic density protein-95; PSF, paroxetine-treated short-time floating; PSMA2, proteasome subunit α type-2; UPS, ubiquitin–proteasome system; VIF, vehicle-treated intermediate-time floating.

To examine whether differential protein expression levels between PLF and PSF mice were caused by transcriptional alterations, we performed quantitative reverse transcription PCR analysis for NMDAR subunits PSD-95 and nNOS (Figure 4a). As we did not see any transcript level differences between the groups, we next examined the possible involvement of ubiquitination-induced proteasomal degradation in the observed protein expression differences. Although no ubiquitinated GluN1, GluN2A and PSD-95 difference was detected between the PLF and PSF mice (Figure 4b), the PSF mice showed greater total proteasome subunit α type-2 (PSMA2) and ubiquitination levels compared with PLF mice (PSMA2: F_2,12_=44.89, *P*<0.0001; ubiquitination: F_2,12_=13.69, *P*<0.001; Figure 4c and Supplementary Figure 2b). Although ubiquitination level in the PLF mice showed significant reduction, its level in PSF was similar to that in control mice.

Taken together, we found significant glutamatergic and ubiquitin–proteasome system (UPS) pathway protein differences between the PLF and PSF groups, further supported by a significant correlation of protein levels with FST immobility time (Supplementary Table 3).

### Glutamatergic and UPS pathway protein regulation in vehicle-treated subgroups

We further investigated glutamatergic and UPS pathway protein expressions in the hippocampus of vehicle-treated subgroups to ascertain whether these protein levels were differentially regulated by FST in the absence of paroxetine treatment (Supplementary Figure 3). We found significant NMDAR subunit expression and phosphorylation level differences between vehicle-treated long-time floating (VLF) and vehicle-treated short-time floating (VSF) groups (P-GluN2A: F_2,12_=79.64, *P*<0.0001; GluN2B: F_2,12_=20.45, *P*<0.0001; P-GluN2B: F_2,12_=19.93, *P*<0.001; Supplementary Figure 3b). Vehicle-treated short-time immobile mice exhibited lower NMDAR subunits and phosphorylation levels than their counterparts, VLF mice. Other glutamatergic and UPS pathway proteins, with the exception of PSD-95, showed no expression level difference between the vehicle-treated subgroups (PSD-95: F_2,12_=30.24, *P*<0.0001; Supplementary Figure 3c). We therefore conclude that NMDAR and PSD-95 expression level differences between VLF and VSF mice are independent of paroxetine treatment.

### Glutamatergic and ubiquitin–proteasome system pathway protein regulation in the prefrontal cortex

Glutamatergic and UPS pathways were also assessed in the prefrontal cortex (Supplementary Figure 4a). We found that prefrontal GluN2A and GluN2B protein levels were significantly lower in PSF compared with PLF mice, whereas none of the prefrontal NMDAR subunit phosphorylation showed a difference. Metabolomics profiles were also investigated in the prefrontal cortex (Supplementary Figure 4b). No significant enrichment was observed for metabolic pathways, and glutamatergic pathway-related metabolites were barely different between the PLF and PSF groups, except for carbamoyl phosphate. Other pathway proteins including nNOS, CAPON and PSMA2 were also found to be differentially regulated between the two groups (Supplementary Figure 4c). Although a significant increase of prefrontal nNOS levels was found in PSF mice, nNOS metabolites including arginine and citrulline were not different between the PLF and PSF groups indicating no change in prefrontal NO pathway activity (Supplementary Figure 4d).

### sGC-β1, PSMA2 and ubiquitin protein expression levels are associated with clinical antidepressant treatment response in PBMCs from MDD patients

**Figure 5:**
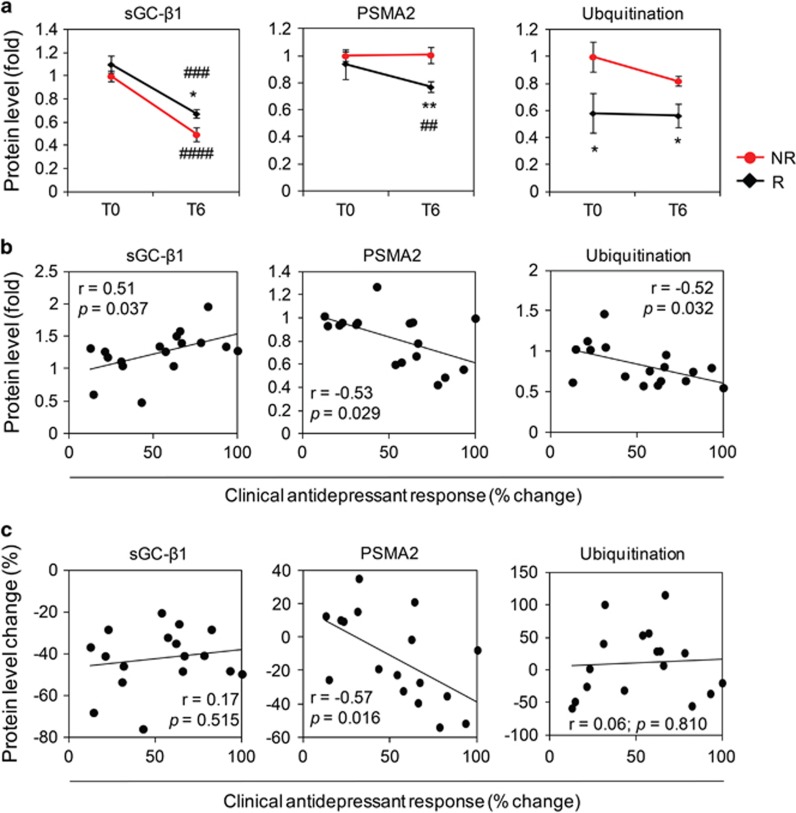
sGC-β1, PSMA2 and ubiquitination levels in human PBMCs from antidepressant responder and non-responder patients. (**a**) sGC-β1, PSMA2 and ubiquitination level differences between antidepressant non-responder (NR) and responder patients (R) at baseline (T0) and after 6 weeks treatment (T6), *n*=17. (**b**) Correlation of protein levels at T6 with clinical antidepressant treatment response, *n*=17. (**c**) Correlation of protein level changes (between T0 and T6) with clinical antidepressant treatment response, *n*=17. *P<0.05, **P<0.01 vs NR (two-tailed *t*-test). ^##^*P*<0.01, ^###^*P*<0.001, ^####^*P*<0.0001 vs T0 (two-tailed paired *t*-test). Data are expressed as the mean±s.e.m. The proteins were analysed using total lysate extract. Pearson correlation coefficients (*r*) with *P*-values are indicated in the correlation graphs. PBMC, peripheral blood mononuclear cell; PSMA2, proteasome subunit α type-2; sGC-β1, soluble guanylate cyclase-β1.

To investigate the relevance of the identified biosignatures, sGC-β1, PSMA2 and ubiquitination levels were analysed in MDD patients' PBMCs (Figure 5, Supplementary Figure 5). All three proteins were differentially expressed between the antidepressant responder and non-responder patient groups, especially 6 weeks after admission (T6; Figure 5a). Although sGC-β1 protein levels were significantly reduced in both groups at T6, PSMA2 protein levels were significantly reduced only in the responder patients' PBMCs at T6. Ubiquitination levels were not altered by chronic antidepressant treatment in either group. However, they were lower in responder compared with non-responder patients. All three proteins level at T6 significantly correlated with clinical antidepressant response (Figure 5b). Only PSMA2 protein level change between baseline (T0) and T6 samples was significantly correlated with the clinical antidepressant treatment response (Figure 5c).

## Discussion

In the present study, we investigated molecular pathway differences between mice that responded and mice that did not respond to chronic paroxetine treatment. PLF mice were classified as non-responders based on their long-time immobility in FST, whereas PSF mice exhibited a significantly shorter FST immobility time and were classified as responders. As the FST is a behavioural test commonly used to evaluate antidepressant-like effects in mice,^21, 22^ we decided to categorize animals based on their FST immobility time. In pilot studies, we investigated whether baseline (pre-treatment) FST impacts on post-treatment FST. Baseline FST outcome did not correlate with post-treatment FST phenotype, suggesting that the heterogeneous FST immobility distribution in paroxetine-treated mice results from chronic paroxetine treatment and is not an inherent behavioural trait of the animal (data not shown). The variability DBA/2 J mice had in the time they spent immobile in the FST at the end of treatment provided a model for investigating the heterogeneous antidepressant treatment response.

To further analyse behavioural differences between the PLF and PSF groups, we have performed female-urine-sniffing test, which has been used to evaluate anhedonic-like behaviour of animals.^23^ Sniffing time differences between the groups showed strong tendency towards statistical significance (*P*=0.052, data not shown).

Our results suggest that proteins and metabolites associated with the glutamatergic pathway are affected by chronic antidepressant treatment in a distinctive way in responders and non-responders. The glutamatergic pathway has previously been associated with MDD pathobiology and antidepressant response. In depressed patients, significantly elevated serum, plasma and cerebrospinal fluid glutamate levels were found.^24, 25, 26, 27, 28^ A single-nucleotide polymorphism in metabotropic 7 glutamate receptor was shown to be involved in the onset of the clinical antidepressant effect.^29^ Glutamate release decreases with chronic fluoxetine, desipramine, reboxetine, venlafaxine or agomelatine treatment.^30, 31^ Numerous studies have shown that chronic antidepressant treatment regulates glutamatergic receptor expression in rodent hippocampus.^32, 33, 34^

Other proteins investigated in the current study, CaMK2,^35^ GDH1,^36, 37^ GSK-3β,^38, 39^ P-ERK,^40, 41, 42^ SYNJ1,^43, 44, 45^ have been associated with MDD pathology and/or antidepressant treatment response.

The NO system has been implicated in the treatment,^22, 46, 47, 48, 49, 50, 51, 52, 53, 54^ as well as the pathobiology of depression.^49, 50, 55, 56, 57^ We, therefore, further investigated PSD-95, nNOS, CAPON and sGC-β1. PSD-95 and nNOS proteins have been shown to associate using PDZ domain-based interaction. They are part of a tertiary complex with NMDAR and produce NO.^58, 59, 60^ CAPON sequesters nNOS and negatively regulates its association with NMDAR/PSD-95 complex.^61^ As CAPON overexpression has been shown to disrupt PSD-95/nNOS complex,^62^ high CAPON levels in PSF mice may prevent catalytic activation of nNOS, which may lead to low levels of citrulline, a product metabolite of nNOS. sGC-β1 is a subunit of soluble guanylate cyclase (sGC) that mediates NO signalling pathways.^63, 64^ sGC-β1 level difference was found significant in post-treatment PBMCs samples from depressed patients implicating the role of NO system in clinical antidepressant response.

Among the investigated proteins, only PSD-95 protein levels were lower in both short-time floating animals (VSF and PSF) compared with their counterparts, long-time floating animals (VLF and PLF). Thus, PSD-95 expression may be affected by FST, and not by chronic paroxetine treatment.

Although NMDAR subunit and phosphorylation level differences were also found significant between VLF and VSF mice, the protein expression pattern was opposite to that found in the PLF and PSF groups suggesting that NMDAR expression and activity was distinctly affected in the absence and presence of chronic paroxetine treatment.

Our results were further corroborated by metabolite profiling data. Although several metabolites including alanine, asparagine, glutamate, glutamine and glutathione did not reach statistical significance after false discovery rate correction, they showed differential levels between the PLF and PSF groups (*P*<0.05), which supports a glutamatergic pathway activity difference between the two groups. Alanine and citrate are known to regulate NMDAR activity.^65, 66^ Glutathione was found to be an NMDAR agonist.^67, 68^ Sarcosine is also an NMDAR co-agonist.^69, 70^ The role of serine as a potent co-agonist for NMDAR has been well demonstrated.^71, 72^ In addition, taurine is suggested to interact directly with NMDAR and regulate its function.^73^ Low serum asparagine, serine and taurine levels characterized non-responder patients after undergoing 5 weeks of antidepressant treatment.^74^ In addition, glutathione, sarcosine and taurine administrations have antidepressant-like effects suggesting their elevated levels might be relevant to the favourable paroxetine response.^68, 70, 75^ Xanthurenic acid was shown to be an agonist for metabotropic glutamate receptor 2 and 3 (mGluR2/3).^76^ These metabolite level differences indicate higher hippocampal glutamatergic activity in PSF compared with PLF mice. As GDH1 catabolizes glutamate, low levels of GDH1 protein might be the cause of the observed elevated glutamate levels in PSF mice. Alternatively, high glutamate levels might induce a compensatory feedback regulation of GDH1 protein expression to prevent pathway overactivation. Altered NMDAR subunit levels are consistent with the observed glutamate levels in the PLF and PSF mice. More glutamate and other glutamatergic receptor modulators could result in reduced NMDAR expression as previously reported in studies with l-trans-pyrrolidine-2,4-dicarboxylate, a high-affinity glutamate reuptake inhibitor.^77, 78^

Our proteomics data derived from the membrane-associated fraction also showed that levels for vesicular glutamate transporter 1, a presynaptic protein that regulates glutamate release, were significantly higher in the PSF than in the PLF mice. This suggests that PSF mice may have a greater glutamate release compared with the PLF mice.

When we investigated NMDAR subunits and PSD-95 protein ubiquitination differences between the PLF and PSF groups, we found that chronic antidepressant treatment induced different regulation of UPS proteins between drug responders and non-responders, both in animals and humans. This is supported by evidence from other studies, which suggests that UPS may be linked to MDD treatment. In this regard, it has been shown that single-nucleotide polymorphisms in proteasome subunit α7 (PSMA7), proteasome 26 S non-ATPase subunit 9 (PSMD9) and proteasome 26 S non-ATPase subunit 13 (PSMD13) are associated with the clinical antidepressant response.^79, 80^ We therefore submit that UPS proteins such as PSMA2 and ubiquitination are of interest for evaluating and predicting clinical antidepressant treatment response.

PSMA2 is an essential subunit that facilitates 20 S core proteasome particle formation that further interacts with 19 S regulatory particle to form 26 S proteasome.^81, 82^ Ubiquitination, covalent conjugation of ubiquitin, acts as a signal to guide proteasome to proteins destined for degradation.^83^ The fact that hippocampal PSMA2 and ubiquitination expression patterns are inconsistent between mouse and human may be due to different tissue sources (hippocampus vs PBMCs). Owing to the limited amount of mouse blood, we were unable to isolate sufficient PBMC material to proof that this is indeed the case. Other proteins related to psychiatric disorders and the antidepressant response including p11 and brain-derived neurotrophic factor have also been shown to be inversely correlated between brain and periphery.^84, 85^ In addition, ubiquitination levels were higher in antidepressant non-responder patients. As stressful events are known to alter the UPS pathway,^86, 87^ that might be a reflection of that.

Interestingly, UPS mediates NMDAR degradation.^88, 89^ Tai *et al.*^90^ reported that NMDA treatment of cultured hippocampal neurons decreases UPS activity, implicating an interplay between glutamatergic and proteasome pathways. This is in agreement with our data, which also point to an involvement of both pathways in the different response of the PLF and PSF mouse groups. Although we failed to find a relationship between ubiquitination and distinct NMDAR subunit protein levels between PLF and PSF mice, other protein degradation mechanisms might cause the observed differential expression. These include ubiquitination-like modifications such as human leuokocyte antigen-F associated transcript 10,^91, 92^ neural precursor cell expressed developmentally downregulated protein 8 (ref. 93) and small ubiquitin-related modifier,^94^ all shown to be involved in proteasomal degradation. In addition, autophagic protein degradation may be relevant for differential protein expression in paroxetine-treated mice.^95, 96^

Although individual housing has been shown to regulate stress response,^97^ Hilakivi *et al.*^98^ have shown that long-term (10–20 days) single housing did not result in a different stress response compared with group-housing in DBA/2 mice. Although the effect of single housing cannot be excluded, FST immobility distribution of the PLF and PSF groups can be the result of chronic paroxetine treatment based on distinct pathway protein expression patterns observed between the vehicle- and paroxetine-treated subgroups.

PSF mice showed similar glutamatergic and UPS pathway protein levels as control mice, whereas PLF mice exhibited significantly different pathway protein levels compared with the other groups. This indicates a dysregulated pathway activity on chronic paroxetine treatment in the PLF mice that is associated with chronic paroxetine treatment non-response.

Heterogeneous FST immobility of vehicle-treated mice may be a reflection of a distinct individual response to FST, an acute stressful behavioural test. As protein expression levels were highly variable in vehicle-treated subgroups, only vehicle-treated intermediate-time floating group mice were selected and used for further analyses.

Our analysis also revealed that chronic paroxetine treatment did not induce significant differences in NMDAR subunit phosphorylation and glutamatergic metabolite levels in the prefrontal cortex. This suggests that there might be closer glutamatergic protein–metabolite interaction in the hippocampus than that in the prefrontal cortex. Although we found a significant nNOS protein level difference, the lack of PSD-95 and citrulline level differences indicates that prefrontal NO system is not differentially affected between the PLF and PSF groups. Significant PSMA2 level difference in the prefrontal cortex further supports PSMA2 protein as a biomarker candidate for the antidepressant response.

Although we were able to identify biomarker candidates that predict and evaluate the clinical antidepressant treatment response, a time-course analysis will be necessary to further corroborate these findings.

In the present study, we used wild-type stress naive DBA2/J mice to explore pharmacological heterogeneity of chronic paroxetine response. An animal model with a depression-like phenotype may provide additional information for the interaction of drug treatment and stress response.

Whether chronic paroxetine treatment response and the identified pathway activities remain in PSF mice after drug discontinuation will be investigated in a future study. An extension of the analysis to microdialysis should also add further information on the glutamate levels and release regulation.

The present study attempts to integrate quantitative proteomics and metabolomics data to improve our understanding of biological pathway changes relevant in the response to antidepressant treatment. What roles glutamatergic and ubiquitin systems have in the underlying mechanism of selective serotonin reuptake inhibitor response requires further studies with compounds able to modulate pathway activity. Pathway modulators may lead to novel drugs with antidepressant activities. In addition, the glutamatergic and ubiquitin systems activities could be used as a biosignature for the antidepressant treatment response.
