# Supplementary material for: Delineation of molecular pathway activities of the chronic antidepressant treatment response suggests important roles for glutamatergic and ubiquitin–proteasome systems
Source: Transl Psychiatry. 2017 Apr 4;7(4):e1078–. doi: 10.1038/tp.2017.39 (PMC5416684; doi:10.1038/tp.2017.39)
Supplement: Supplementary Information [file tp201739x1.doc]

**Supplemental Materials and Methods**

**Mouse Brain and Blood Collection**

Blood was collected at least one month before commencing paroxetine treatment from retro-orbital puncture and after 28 days of paroxetine treatment through cardiac puncture or trunk blood. On day 29, the animals were subjected to the FST 4 hours after the last administration of paroxetine and sacrificed immediately after the test. Trunk blood and brains of the animals were collected and stored at -80°C until further use. Blood was centrifuged to separate plasma and erythrocytes (1300g, 10 min, 4°C) before storage.

**Proteomics Analysis**

To isolate cytoplasm-associated fraction, mouse hippocampi were homogenized in a buffer containing 2M NaCl, 10mM HEPES/NaOH, 1mM EDTA and protease inhibitor cocktail tablets (Roche Diagnostics, Mannheim, Germany) and phosphatase inhibitors (Sigma, St. Louis, MO, USA). Homogenates were sonicated with an ultra-sonicator (Branson, Danbury, CT, USA) and then centrifuged (16100 g, 20 min, 4°C). In the cytoplasm-associated fraction, the protein concentration was determined by Bradford assay. For the membrane-associated fraction, the pellet was processed as previously described ^1^. Briefly, the pellet was resuspended in a buffer containing 0.1M Na_2_CO_3_ and 1mM EDTA and then centrifuged (16100g, 20 min, 4°C). Further resuspension was performed with a buffer containing 1mM EDTA, 10mM HEPES, 100mM NaCl, 5M Urea. After additional pellet resuspension with a buffer containing 0.1M Tris/HCl, the pellet was centrifuged (16100 g, 20 min, 4°C) and dissolved in a buffer containing 2% SDS, 50mM DTT, 0.1M Tris/HCl, protease inhibitor cocktail tablets (Roche Diagnostics, Mannheim, Germany) and phosphatase inhibitors (Sigma, St. Louis, MO, USA). Lowry assay was used to quantify the protein concentration in the membrane-associated fraction using DC Protein Assay kit (Bio-Rad Laboratories, Munich, Germany).

Protein extracts were mixed with equal amounts of ^15^N-labeled DBA/2 mouse hippocampal protein extract ^2^. Fourty µg of the protein mixture was separated in a 10% SDS-PAGE gel and stained with Coomassie Brilliant Blue R-250 (BioRad, Hercules, CA, USA) followed by overnight destaining. After cutting the gel lane into slices, tryptic peptides were produced and extracted as previously described ^3^. Extracted peptides were analyzed by liquid chromatography-electrospray tandem mass spectrometry (LC-MS/MS) using a nanoflow HPLC-2D system (Eksigent, Dublin, California) coupled online to an LTQ-Orbitrap mass spectrometer (Thermo Fisher Scientific, Bremen, Germany). Protein identification and quantitation were performed as described previously ^4^. The list of proteins differentially expressed between PLF and PSF groups is presented in Supplementary Table 2.

**Metabolomics Analysis**

Mouse brain and plasma metabolites were extracted and analyzed with targeted metabolomics as previously described ^5^. A 30-fold excess (w/v) of 80% cold methanol was added to the hippocampus and prefrontal cortex. Brain tissues were homogenized (1200 min^-1^, 2 min, Potter-S homogenizer, Sartorius, Göttingen, Germany) on ice and centrifuged (14000 g, 10 min, 4°C). Supernatants were transferred and a 6-fold excess (w/v) of 80% cold methanol was added to the pellets. Pellets were sonicated to further extract metabolites and combined with the previous supernatants. Combined samples were vortexed, centrifuged (14000 g, 10 min, 4°C), and lyophilized. Mouse plasma metabolites were extracted with a 4-fold excess (v/v) of 100% cold methanol. After vortexing for 2 min, samples were incubated on dry ice for 2 h and centrifuged (2053 g, 100 min, 4°C). Supernatants were filtered using a 0.22 μm ultrafiltration tube (1105g, 2 min, 4°C) and the filtrates were lyophilized. The lyophilized metabolites were stored at -80°C until further use. Samples were dissolved in 20 μl liquid chromatography-mass spectrometry grade water. Ten microliters were injected and analyzed using a 5500 QTRAP triple quadrupole mass spectrometer (AB/SCIEX, Framingham, MA, USA) coupled to a Prominence UFLC high-performance liquid chromatography system (Shimadzu, Columbia, MD, USA) via selected reaction monitoring of a total of 280 endogenous water-soluble metabolites for steady-state analyses of samples. Samples were delivered to the mass spectrometer via normal phase chromatography using a 4.6-mm i.d × 10 cm Amide Xbridge HILIC column (Waters, Milford, MA, USA) at 350 μl min^−1^. Gradients were run starting from 85% buffer B (high-performance liquid chromatography grade acetonitrile) to 42% B from 0 to 5 min; 42% B to 0% B from 5 to 16 min; 0% B was held from 16 to 24 min; 0% B to 85% B from 24 to 25 min; 85% B was held for 7 min to re-equilibrate the column. Buffer A comprised 20mM ammonium hydroxide/ 20mM ammonium acetate (pH = 9.0) in 95:5 water:acetonitrile. Some metabolites were targeted in both positive and negative ion modes for a total of 291 selected reaction monitoring transitions using positive/negative polarity switching. Electrospray ionization voltage was +4900 V in positive ion mode and − 4500 V in negative ion mode. The dwell time was 4ms per selected reaction monitoring transition and the total cycle time was 1.89 s. Approximately 9–12 data points were acquired per detected metabolite. Peak areas from the total ion current for each metabolite-selected reaction monitoring transition were integrated using the MultiQuant v2.0 software (AB/SCIEX, Darmstadt, Germany). Animals from the same cohort were used for all metabolomics analyses. The list of metabolites measured is presented in Supplementary Table 2.

**Functional Enrichment Analysis**

Proteomics pathway enrichment was performed using DAVID bioinformatics resources 6.7 according to a Kyoto Encyclopedia of Genes and Genomes (KEGG) database ^6^. All genes encoded by the genome were used as default background. Enriched pathways were considered significant at Bonferroni adjusted *p* < 0.05 and FDR < 0.01. Proteins common to several pathways were further extracted from the enriched pathways using a Venn diagram comparison. The protein interaction network was created using STRING database.

**Western Blot Analysis**

Mouse hippocampi and prefrontal cortices were homogenized with the same buffer used for proteomics sample preparation, or RIPA buffer containing protease inhibitor cocktail tablets (Roche Diagnostics, Mannheim, Germany) and phosphatase inhibitors (Sigma, St. Louis, MO, USA). Patient PBMCs were homogenized with RIPA buffer containing protease and phosphatase inhibitors. Homogenates were sonicated and centrifuged (10000 g, 20 min, 4°C). Bradford assay was used to quantify extracted protein concentration. Proteins were separated in a 10-15% gradient SDS-PAGE gel. Subsequently, they were transferred to a PVDF membrane (Millipore, Billerica, MA, USA). After blotting, the membrane was blocked with 5% skim milk solution for 1 h at room temperature and incubated with either Ca^2+^/calmodulin-dependent protein kinase (CaMK) 2 (ab50202, 1:2000, Abcam, Cambridge, UK), carboxy-terminal PDZ ligand of nNOS (CAPON) (sc-8532, 1:500, Santa Cruz, Dallas, TX, USA), extracellular signal-regulated kinase (ERK) (#9102, 1:1000, Cell Signaling, Danvers, MA, USA), glutamate dehydrogenase 1 (GDH1) (ARP45709_P050, 1:1000, Aviva System Biology, San Diego, CA, USA), glutamine synthetase (GS) (HPA007316, 1:1000, Sigma, St.Louis, MO, USA), glycogen synthase kinase-3β (GSK-3β) (#9315, 1:1000, Cell Signaling, Danvers, MA, USA), soluble guanylate cyclase-β1 (sGC-β1) (sc-20955, 1:500, Santa Cruz, Dallas, TX, USA), mitochondrial aspartate transaminase (mAST) (sc-46704, 1:500, Sigma, St.Louis, MO, USA), mitogen-activated protein kinase kinase (MEK) (#9122, 1:1000, Cell Signaling, Danvers, MA, USA), neuronal nitric oxide synthase (nNOS) (#4236, 1:1000, Cell Signaling, Danvers, MA, USA), N-Methyl-D-aspartate receptor (NMDAR) subunit GluN1 (sc-1467, 1:500, Santa Cruz, Dallas, TX, USA), NMDAR subunit GluN2A (sc-1468, 1:500, Santa Cruz, Dallas, TX, USA), NMDAR subunit GluN2B (sc-1469, 1:500, Santa Cruz, Dallas, TX, USA), phospho-CaMK2 (P- CaMK2) (#3361, 1:1000, Cell Signaling, Danvers, MA, USA), phospho-ERK (P-ERK) (#4377, 1:1000, Cell Signaling, Danvers, MA, USA), phospho-GSK-3β (P-GSK-3β) (#9336 ,1:1000, Cell Signaling, Danvers, MA, USA), phospho-MEK (P-MEK) (#9121, 1:1000, Cell Signaling, Danvers, MA, USA), phospho-GluN1 (P-GluN1) (ab68144, 1:500, Abcam, Cambridge, UK), phosphor-GluN2A (P-GluN2A) (#4206, 1:1000, Cell Signaling, Danvers, MA, USA), phospho-GluN2B (P-GluN2A) (#5355, 1:1000, Cell Signaling, Danvers, MA, USA), postsynaptic density protein-95 (PSD-95) (#75-028, 1:1000, NeuroMab, Irvine, CA, USA), proteasome subunit α type-2 (PSMA2), synapsin (#2312, 1:1000, Cell Signaling, Danvers, MA, USA), synaptic vesicle glycoprotein 2A (SV2A), synaptojanin 1 (SYNJ1), syntaxin binding protein1 (STXBP1) or ubiquitin (Ub) (sc-8017, 1:500, Santa Cruz, Dallas, TX, USA) antibody at 4°C overnight. PSMA2, SYNJ1, STXBP1 and SV2A antibodies were provided by the Human Protein Atlas (HPA) program (Albanova University Center, Royal Institute of Technology, Sweden).

The membranes were washed and then incubated with horseradish peroxidase (HRP) conjugated-secondary antibodies. The blots were developed with Luminata^TM^ Forte Western HRP Substrate (Millipore, Billerica, MA, USA). Images were acquired by ChemiDoc^TM^ MP imaging system (Bio-Rad Laboratories, Munich, Germany). Densitometric data analyses were carried out with ImageJ software (National Institute of Health, USA). Protein expression levels were normalized with total protein, which was quantified from the Coomassie blue-stained membrane lanes.

**Quantitative Reverse Transcription Polymerase Chain Reaction (qRT-PCR)**

Hippocampal total RNA was isolated with TRIzol reagent (Invitrogen, Karlsruhe, Germany) as previously described ^7^. RNA levels were quantified using NanoPhotometer (IMPLEN, Munich, Germany). One ug of RNA was subjected to reverse transcription using Omniscript RT kit according to manufacturer’s protocol (Quiagen, Santa Clarita, CA, USA). QuantFast SYBR Green PCR kit (Quiagen, Santa Clarita, CA, USA) was used for qRT-PCR. The reaction was performed using LightCycler 480 (Roche Diagnotics, Penzberg, Germany). The cycling condition used was as follows: denaturation step at 95°C for 10min, followed by 45 cycles of amplification step (95°C for 10sec, 60°C for 30sec, for each cycle). Each set of primers was used for detection of glyceraldehyde-3-phosphate dehydrogenase (GAPDH), nNOS, GluN1, GluN2A, GluN2B and PSD-95 (Eurofins MWG Operon, Ebersberg, Germany) (Supplementary Table 1). Each sample was analyzed in duplicate and normalized with GAPDH level. Relative quantitation was performed based on crossing points (CP) value ^8^.

**Immunoprecipitation**

Hippocampal proteins were extracted and immunoprecipitated using Pierce Direct IP kit (Thermo Fisher Scientific, Rockford, IL, USA). Mouse hippocampi were homogenized with IP lysis/wash buffer containing 2% SDS (Sigma, St. Louis, MO, USA), protease inhibitor cocktail tablets (Roche Diagnostics, Mannheim, Germany) and phosphatase inhibitors (Sigma, St. Louis, MO, USA). Lowry assay was performed to measure protein concentration with DC Protein Assay kit (Bio-Rad Laboratories, Munich, Germany). Ten ug of Ub antibody (Santa Cruz, Dallas, TX, USA) was covalently bound to the resin according to manufacturer’s protocol. Five hundred ug of hippocampal lysates were boiled (95°C, 10 min) and diluted with 5 volumes of IP lysis/wash buffer containing 2% Triton X-100, protease inhibitor cocktail tablets (Roche Diagnostics, Mannheim, Germany) and phosphatase inhibitors (Sigma, St. Louis, MO, USA). The lysates were incubated with Ub antibody-coupled resin (4°C, overnight). The resin was washed three times with IP lysis/wash buffer and was boiled with 1×SDS loading buffer for elution (95°C, 10 min). Immunoprecipitates were separated in a 10% SDS-PAGE gel, and Western blot analysis was performed with GluN1, GluN2A and PSD-95 antibodies. Ubiquitinated protein levels were normalized by total ubiquitination intensity.

References

1. Lu A, Wiśniewski JR, Mann M. Comparative proteomic profiling of membrane proteins in rat cerebellum, spinal cord, and sciatic nerve. J. Proteome Res. 2009 May;8(5):2418–25.

2. Zhang Y, Filiou MD, Reckow S, Gormanns P, Maccarrone G, Kessler MS, et al. Proteomic and metabolomic profiling of a trait anxiety mouse model implicate affected pathways. Mol. Cell Proteomics. 2011 Dec.;10(12):M111.008110.

3. Frank E, Kessler MS, Filiou MD, Zhang Y, Maccarrone G, Reckow S, et al. Stable isotope metabolic labeling with a novel N-enriched bacteria diet for improved proteomic analyses of mouse models for psychopathologies. PLoS ONE. 2009;4(11):e7821.

4. Webhofer C, Gormanns P, Reckow S, Lebar M, Maccarrone G, Ludwig T, et al. Proteomic and metabolomic profiling reveals time-dependent changes in hippocampal metabolism upon paroxetine treatment and biomarker candidates. J Psychiatr Res. 2013 Mar.;47(3):289–98.

5. Weckmann K, Labermaier C, Asara JM, Müller MB, Turck CW. Time-dependent metabolomic profiling of Ketamine drug action reveals hippocampal pathway alterations and biomarker candidates. Transl Psychiatry. 2014;4:e481.

6. Huang DW, Sherman BT, Tan Q, Kir J, Liu D, Bryant D, et al. DAVID Bioinformatics Resources: expanded annotation database and novel algorithms to better extract biology from large gene lists. Nucleic Acids Res. 2007 Jul.;35(Web Server issue):W169–75.

7. Schmidt MV, Scharf SH, Sterlemann V, Ganea K, Liebl C, Holsboer F, et al. High susceptibility to chronic social stress is associated with a depression-like phenotype. Psychoneuroendocrinology. 2010 Jun.;35(5):635–43.

8. Pfaffl MW. A new mathematical model for relative quantification in real-time RT-PCR. Nucleic Acids Res. 2001 May 1;29(9):e45.

**Supplemental Legends**

**
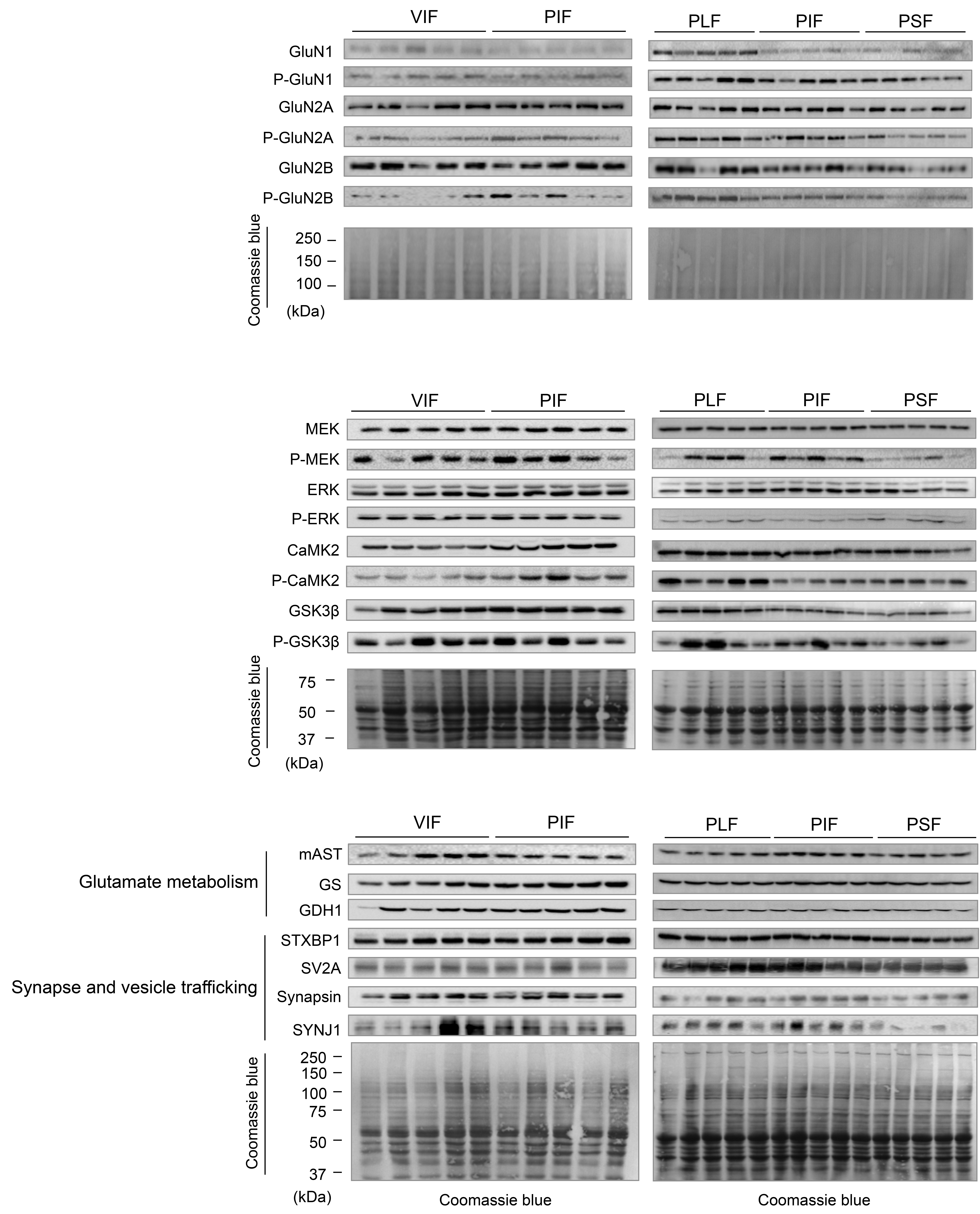
**

**Supplementary Figure 1**. Representative Western blots of Figure 2. NMDAR subunits were blotted from membrane-associated fraction of hippocampus. The rest proteins were blotted using cytoplasm-associated fraction of hippocampus. Comassie Brilliant Blue staining was used as a loading control. PIF group was used as a standard to compare with control, vehicle-treated intermediate floating (VIF), group.

**
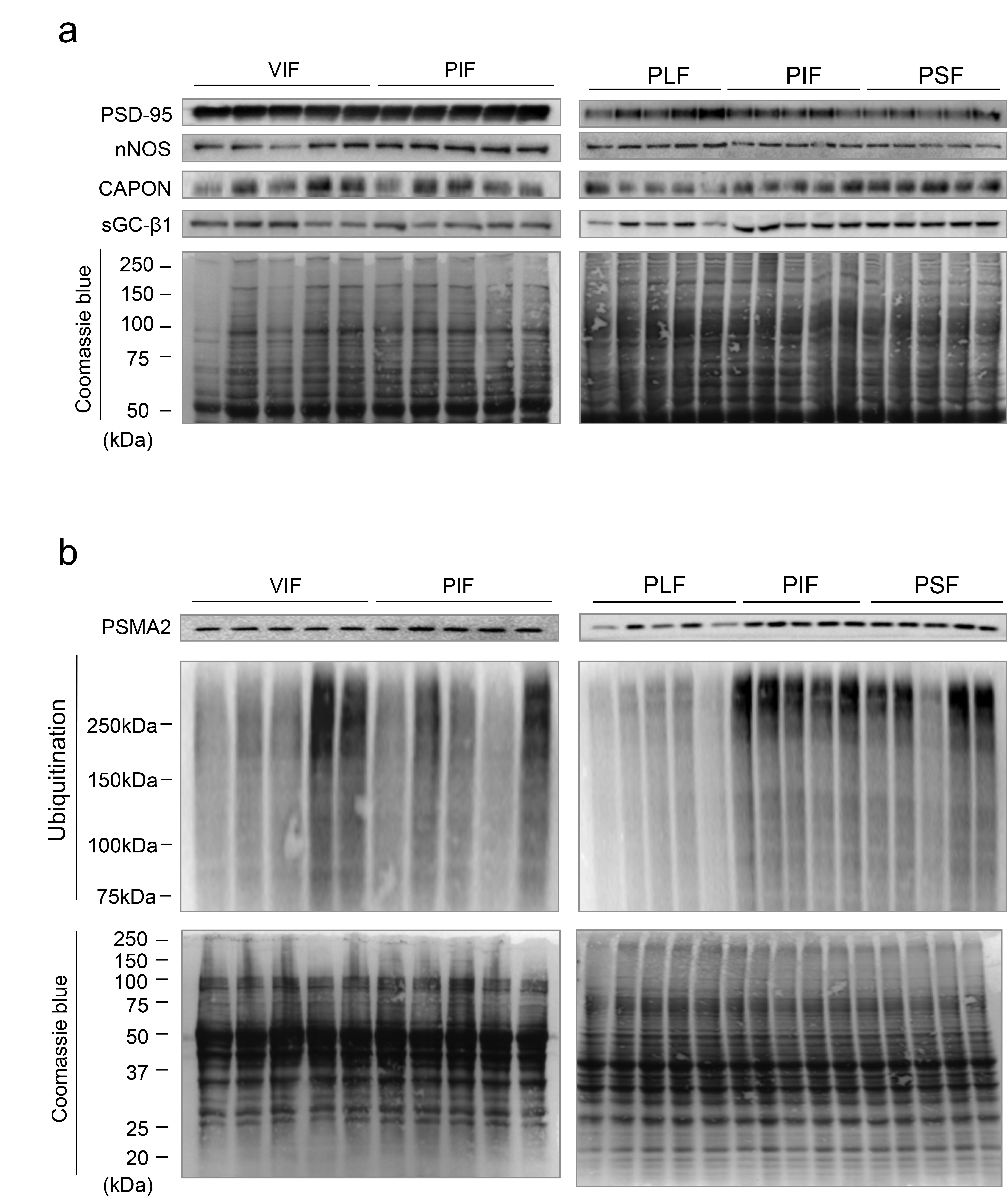
**

**Supplementary Figure 2.** Representative Western blots of Figure (**a**) 3a and (**b**) 4c. The proteins were blotted using cytoplasm-associated fraction of hippocampus. Comassie Brilliant Blue staining was used as a loading control. PIF group was used as a standard to compare with control (VIF) group.


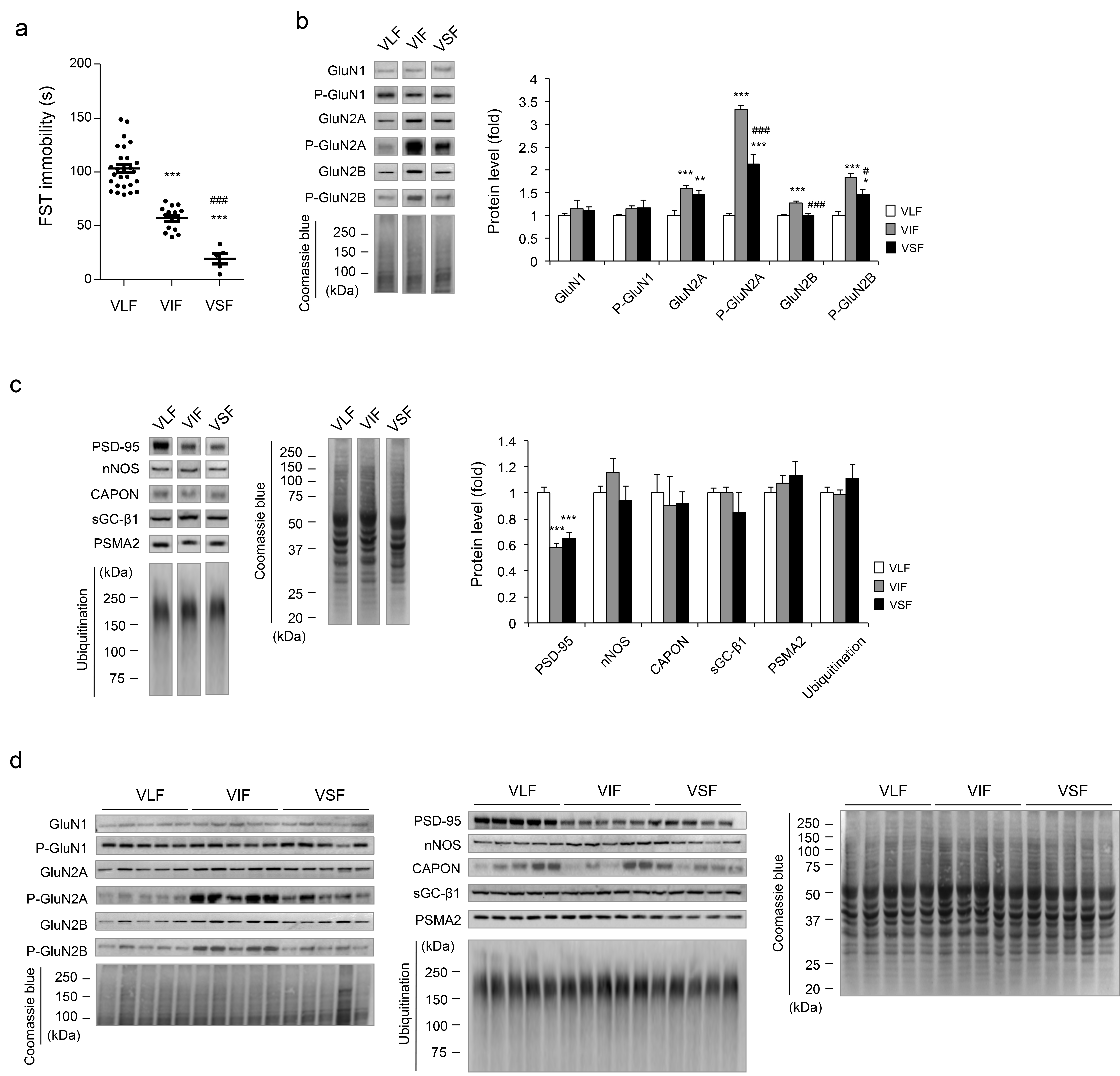


**Supplementary Figure 3**. Glutamatergic and UPS pathway protein expressions in the hippocampus of vehicle-treated sub-groups. (**a**) Vehicle-treated mice were categorized into vehicle-treated long floating (VLF), vehicle-treated intermediate floating (VIF) and vehicle-treated short floating (VSF) groups based on FST immobile time, *n*(VLF/VIF/VSF)*=27*/15/5. (**b**) NMDAR subunits protein and phosphorylation levels among the groups. The proteins were blotted using membrane-associated fraction, *n=*5/group. (**c**) PSD-95, nNOS, CAPON, sGC-β1, PSMA2 and ubiquitination protein level differences among the groups, *n=*5/group. The proteins were blotted using cytoplasm-associated fraction. (**d**) Representative Western blots of the pathway. Data are expressed as the mean ± SEM. **p* < 0.05, ***p* < 0.01, ****p* < 0.001 vs. VLF, ^#^*p* < 0.05, ^###^*p* < 0.001 vs. VIF (one-way ANOVA with Tukey’s test). Comassie Brilliant Blue staining was used as a loading control.


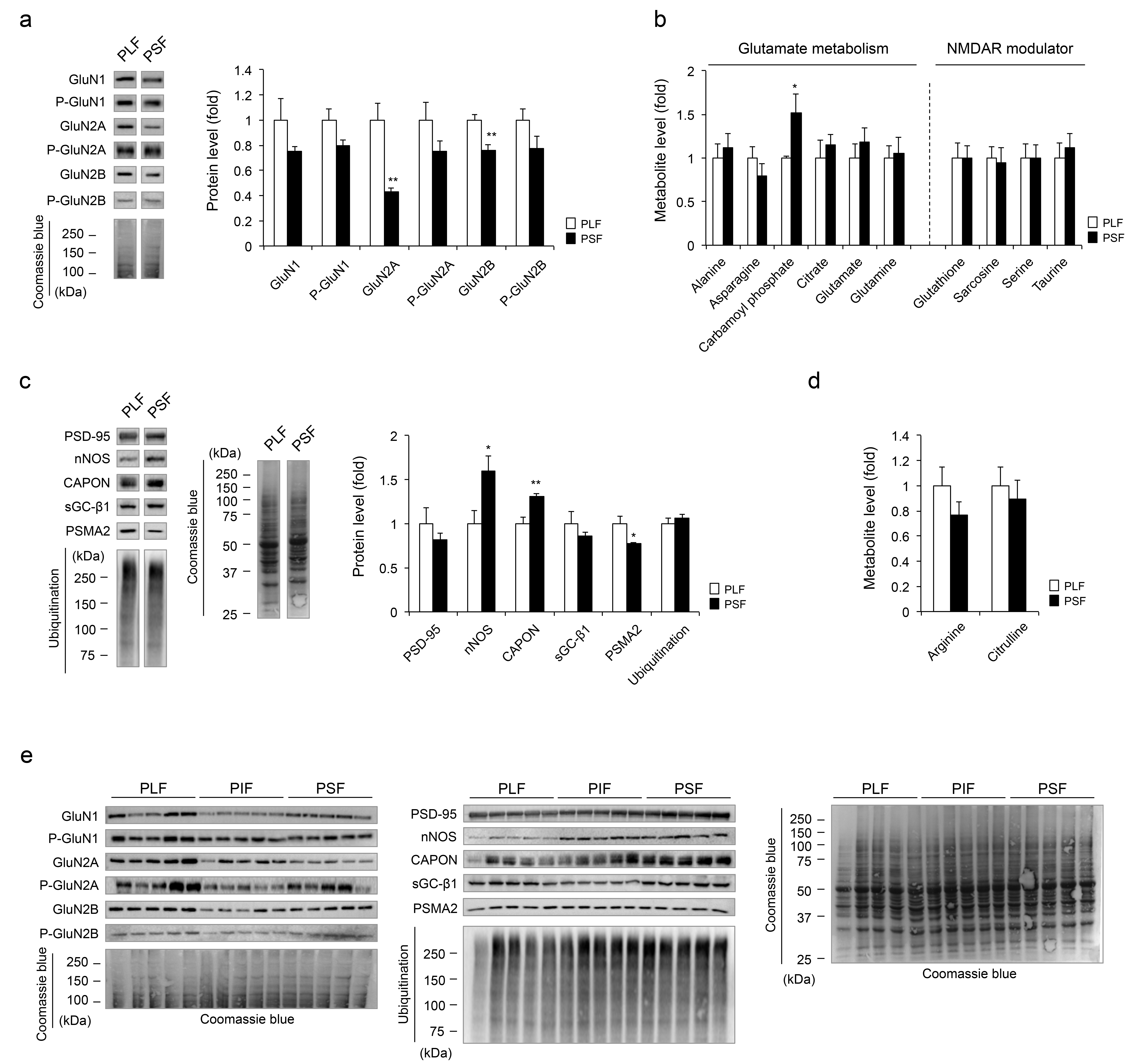


**Supplementary Figure 4**. Glutamatergic and UPS pathway differences between the PLF and PSF mice in the prefrontal cortex. (**a**) NMDAR subunits protein and phosphorylation levels among the groups, *n=*5/group. The proteins were blotted using membrane-associated fraction. (**b**) Glutamate pathway-related metabolite differences between PLF and PSF mice, *n=*5/group. (**c**) PSD-95, nNOS, CAPON, sGC-β1, PSMA2 and ubiquitination protein level differences among the groups, *n=*5/group. The proteins were blotted using cytoplasm-associated fraction. (**d**) Arginine and citrulline levels in PLF and PSF mice, *n=*5/group. (**e**) Representative Western blots of the pathway proteins. Data are expressed as the mean ± SEM. **p* < 0.05, ***p* < 0.01 vs. PLF (two tailed *t*-test). Comassie Brilliant Blue staining was used as a loading control.


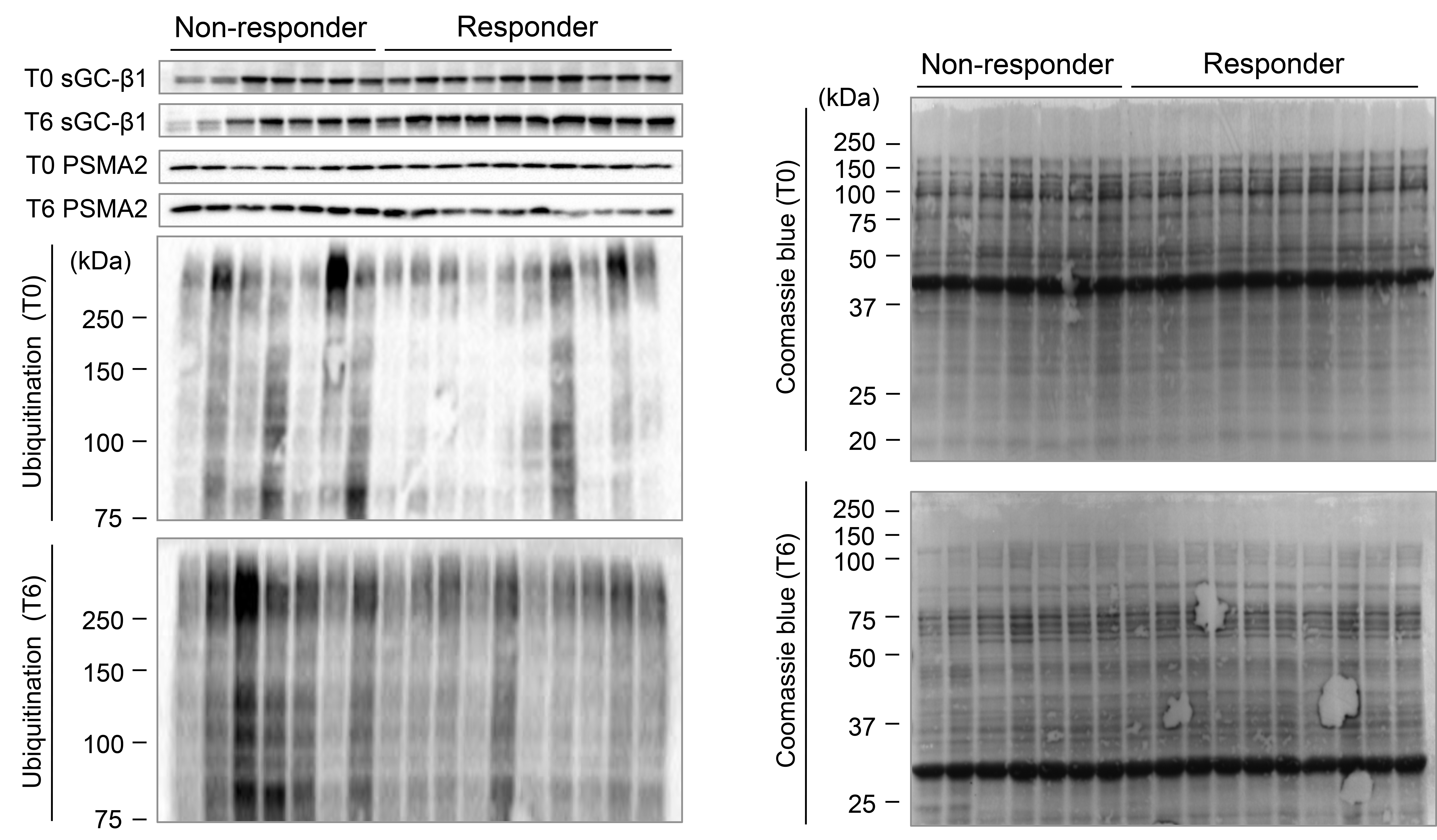


**Supplementary Figure 5**. Representative Western blots for Figure 5.


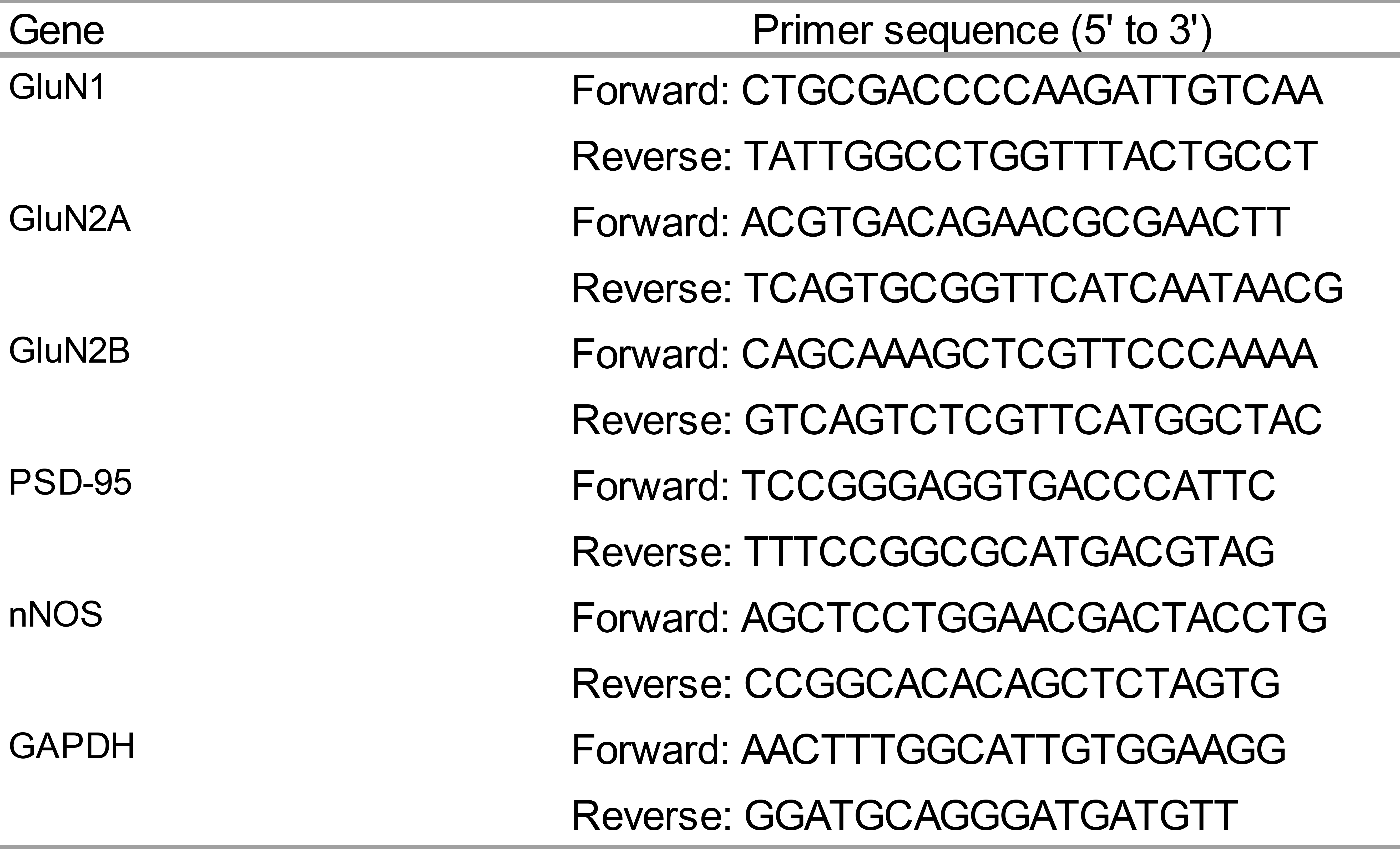


**Supplementary Table 1**. List of primers used for qRT-PCR analysis.

**Supplementary Table 2.** Quantified proteins and metabolites of PLF and PSF groups (*n=5* per group).


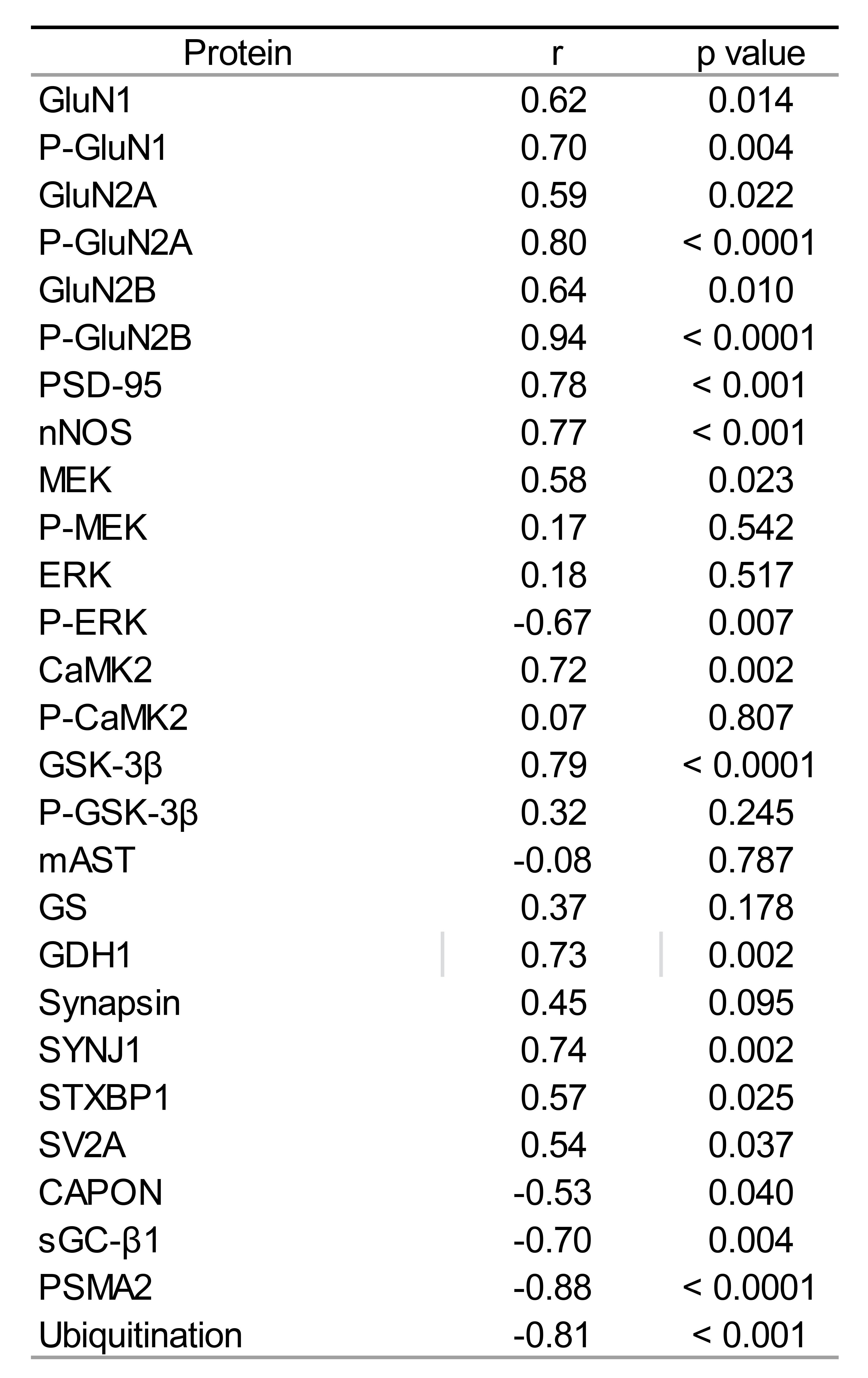


**Supplementary Table 3**. Spearman correlation of hippocampal pathway protein levels with FST immobile time in paroxetine-treated mice, *n=1*5.


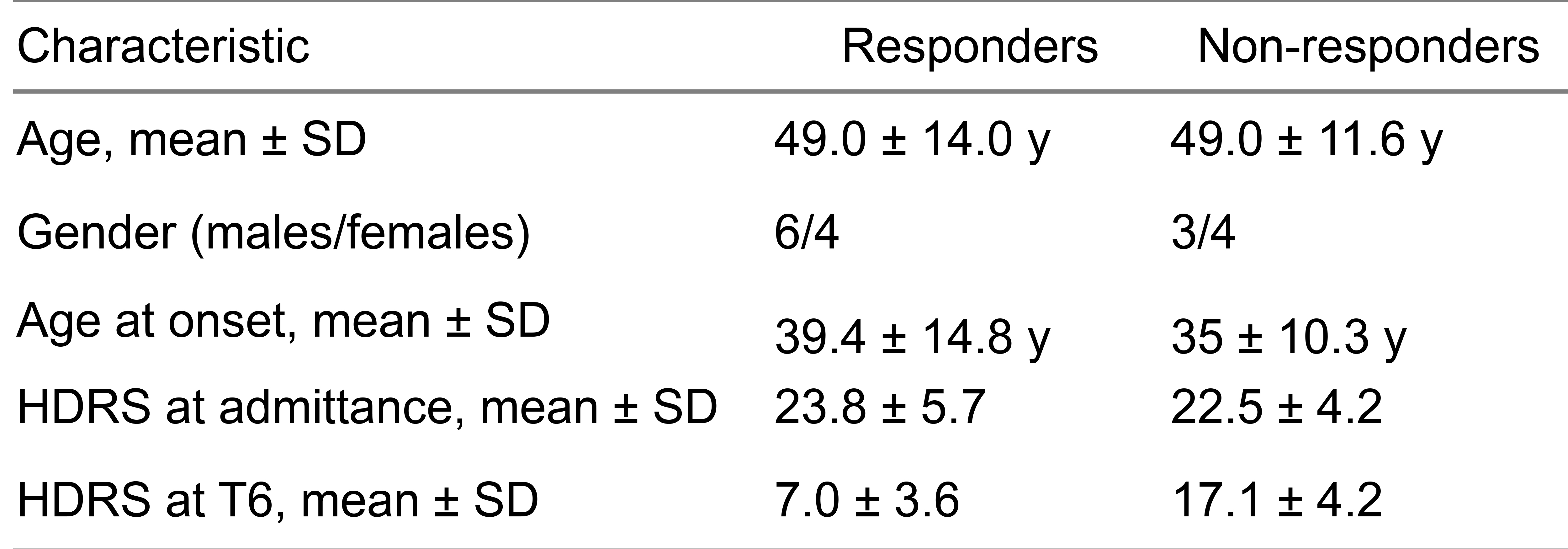


**Supplementary Table 4**. Demographic features of antidepressant treatment responder and non-responder patients.
